# Impact of Smart Hearing Aid Technology on Self-Esteem in Patients with Sensorineural Hearing Loss: A Cross-Sectional Multivariate Study

**DOI:** 10.3390/healthcare14101336

**Published:** 2026-05-13

**Authors:** Liviu Lucian Padurean, Mirela Frandes, Horatiu Eugen Stefanescu, Calin Muntean, Ioana Delia Horhat

**Affiliations:** 1 Doctoral School, “Victor Babeș” University of Medicine and Pharmacy, 300041 Timișoara, Romania; lucian.padurean@umft.ro; 2 Department of Otorhinolaryngology, Municipal Clinical Hospital, 335700 Orăștie, Romania; 3 Department of Functional Sciences—Medical Informatics and Biostatistics, “Victor Babeș” University of Medicine and Pharmacy, 300041 Timișoara, Romania; cmuntean@umft.ro; 4 Center for Modeling Biological Systems and Data Analysis, “Victor Babeș” University of Medicine and Pharmacy, 300041 Timișoara, Romania; 5 Department IX, Discipline of Otorhinolaryngology, “Victor Babeș” University of Medicine and Pharmacy, 300041 Timișoara, Romania; horhat.ioana@umft.ro

**Keywords:** hearing aids, smart technologies, sensorineural hearing loss, self-esteem, Rosenberg Self-Esteem Scale, hearing handicap, tinnitus, psychosocial well-being, neuro-sensory rehabilitation, assistive devices

## Abstract

**Background:** Sensorineural hearing loss represents a significant global health burden affecting over 1.5 billion individuals worldwide. Modern hearing aids, equipped with digital signal processing and smart connectivity features, constitute a cornerstone of neuro-sensory rehabilitation. However, the psychosocial impact of these assistive smart technologies on patient self-esteem remains incompletely characterized. **Methods:** A cross-sectional multivariate study was conducted with 245 participants, divided into three groups: normal-hearing controls (NH, n = 73), hearing-impaired patients using smart hearing aid technology (HA users, n = 86), and hearing-impaired patients not using hearing aid technology (HA non-users, n = 86). Self-esteem was measured using the Rosenberg Self-Esteem Scale (SES). Hearing disability and tinnitus severity were assessed with the Hearing Handicap Inventory for Adults (HHIA) and Tinnitus Handicap Inventory (THI), respectively. Data analysis included one-way ANOVA, Tukey’s HSD post hoc tests, Pearson correlations, and multivariate regression. **Results:** Hearing aid users showed significantly higher SES scores (35.41 ± 5.32) compared to non-users (22.99 ± 4.53; *p* < 0.001, Cohen’s d = 2.515). One-way ANOVA indicated highly significant differences among groups (F = 299.00, *p* < 0.001, η^2^ = 0.712). SES was negatively correlated with HHIA (r = −0.573, *p* < 0.001) and THI (r = −0.443, *p* < 0.001), while HHIA and THI were strongly positively correlated (r = 0.729, *p* < 0.001). In multivariate analysis, HA use remained a strong independent predictor of self-esteem (β ≈ 11.9, *p* < 0.001), even after adjustment for age, sex, HHIA, and THI. Perceived hearing handicap was independently associated with lower self-esteem, whereas tinnitus severity was not a significant predictor in the fully adjusted model. The model explained approximately 65% of the variance in self-esteem scores. **Conclusions:** Smart hearing-aid use is strongly and independently associated with higher self-esteem in patients with sensorineural hearing loss. These results support the inclusion of modern audiological rehabilitation devices in comprehensive management strategies for long-term conditions and highlight psychosocial benefits that extend beyond hearing restoration.

## 1. Introduction

Sensorineural hearing loss (SNHL) is one of the most prevalent disabling conditions worldwide, affecting about 1.5 billion people, according to the World Health Organization [1]. SNHL results from damage to the cochlear hair cells, the auditory nerve, or the central auditory pathways. Unlike conductive hearing loss, SNHL is generally permanent and cannot be medically or surgically reversed. In children, undetected or untreated SNHL impairs language acquisition and academic attainment without early intervention services, while in adults it progressively disrupts communication, social engagement, and quality of life [2]. The global burden of hearing loss is expected to increase significantly, with estimates indicating that by 2050, nearly 2.5 billion individuals will experience some degree of hearing impairment, underscoring the need for effective management strategies [3].

Self-esteem, defined as an individual’s subjective assessment of their own worth, is a core psychological concept closely tied to mental health outcomes [4]. Low self-esteem has consistently been linked to depression, anxiety, and reduced social functioning [5]. In the context of SNHL, the link between auditory disability and psychological well-being has attracted growing scholarly interest, with emerging evidence indicating that untreated hearing impairment may lead to lower self-perception and emotional distress [6,7]. Recent randomized evidence from the ACHIEVE trial further demonstrated that structured hearing intervention yields measurable non-auditory benefits in older adults with hearing loss [8], and the 2024 Lancet Commission report identifies hearing loss as one of the most important modifiable factors across the life course [9]. Tinnitus is a frequent comorbidity of SNHL and has itself been linked to emotional distress, anxiety, and depression; it therefore constitutes a potential confounder of the relationship between hearing status and psychological outcomes and was measured in the present study as an adjustment variable.

Modern hearing aids have evolved from simple acoustic amplifiers into advanced smart devices that incorporate digital signal processing, directional microphones, noise-reduction algorithms, and wireless connectivity [10]. These advances place contemporary hearing aids within the broader ecosystem of wearable health technologies and smart assistive devices that support the management of long-term conditions through ongoing biometric monitoring and personalized interventions [11]. The addition of Bluetooth connectivity, smartphone apps, and AI-driven features has transformed hearing aids into comprehensive neuro-sensory rehabilitation platforms [12].

Despite the recognized audiological benefits of hearing amplification, the psychological effects of hearing aid use—especially on self-esteem—remain poorly understood in the literature [13]. Previous studies have primarily focused on auditory outcomes, speech recognition, and overall quality of life, with limited attention to self-perception and emotional health [14]. Additionally, the relationship among the severity of hearing loss, the co-occurrence of tinnitus, and psychological outcomes associated with hearing aid adoption warrants further clarification [15].

The relationship between hearing rehabilitation and psychological outcomes is likely multifactorial. Self-esteem in patients with SNHL may be influenced not only by device use itself but also by age, sex, perceived hearing handicap, tinnitus severity, and a range of socio-economic and identity-related factors, including communication quality, social support, employment, and deaf cultural identification. Examining this relationship using multivariate analytical approaches is therefore essential to better understand the independent contribution of smart hearing-aid technology while acknowledging that self-esteem remains determined by many other factors not directly captured in audiological assessment.

This study aims to assess the relationship between hearing-aid use and self-esteem among adults with SNHL, using the standardized Rosenberg Self-Esteem Scale (SES) [16]. Secondary goals include analyzing the relationships among self-esteem, perceived hearing handicap (HHIA), and tinnitus severity (THI), thereby providing a comprehensive view of the psychosocial aspects of audiological rehabilitation. We hypothesize that patients using smart hearing-aid technology will have significantly higher self-esteem scores than those without such devices, supporting the inclusion of these devices in long-term condition management plans.

## 2. Materials and Methods

### 2.1. Study Design and Ethical Considerations

This cross-sectional multivariate study was conducted at the Department of Otorhinolaryngology at “Victor Babeș” University of Medicine and Pharmacy in Timisoara, Romania. The investigation was conducted in accordance with the Declaration of Helsinki and received ethical approval from the institutional Ethics Committee (Protocol No. 70/01.11.2023). Written informed consent was obtained from all participants before enrollment.

### 2.2. Participants and Study Cohorts

A total of 245 participants were recruited from specialized otolaryngology clinics and divided into three groups based on hearing status and hearing-aid use: a group of normal-hearing controls (NH), n = 73, with audiometrically confirmed normal hearing thresholds (pure-tone average ≤ 25 dB HL); a group of users with hearing aids (HA users), n = 86, consisting of adults with moderate-to-severe SNHL who have used digital hearing aids for at least six months; and a group of participants without hearing aids (HA non-users), n = 86, with moderate-to-severe SNHL who chose not to use hearing aids for a variety of reasons—including financial constraints, cosmetic or cultural concerns, perceived limited benefit from previous trials, or personal preference—rather than a categorical refusal of clinical advice.

Socio-demographic characteristics of the cohort were extracted retrospectively from clinical records. All participants lived in rural areas of western Romania, and all hearing-aid recipients (n = 86) received their devices through the Romanian National Health Insurance System, consistent with eligibility criteria for publicly funded hearing rehabilitation in this region. Educational attainment was predominantly secondary, with 94.7% of the cohort having completed high school as their highest documented education. A minority (5.3%, n = 13) held a higher educational qualification corresponding to a professional occupation (engineer, pharmacist, firefighter, or police officer). Occupational status reflected the cohort’s age structure: 60.0% (n = 147) were retired, and 40.0% (n = 98) were professionally active. The distribution was strongly skewed toward retirement in both SNHL groups (≥81%), with a corresponding predominance of active workers among the normal-hearing controls (94.5%).

Inclusion criteria included adults aged 18 years or older with stable hearing thresholds and the ability to complete self-administered questionnaires. Exclusion criteria included conductive hearing loss, active middle-ear disease, cognitive impairment, and psychiatric conditions that could independently influence self-esteem assessment. Patients with profound SNHL (pure-tone average > 90 dB HL) were excluded because cochlear implantation, rather than acoustic amplification, is the standard of care in that group and would render the HA-user versus HA-non-user comparison uninformative.

In this study, “smart hearing-aid technology” refers to digital, programmable, multi-channel behind-the-ear or receiver-in-canal devices that, at a minimum, incorporate digital signal processing with automatic environmental classification, adaptive directional microphones, adaptive noise reduction, feedback cancellation, and wireless connectivity (Bluetooth Low Energy or proprietary 2.4-GHz protocols) paired with dedicated smartphone applications for user control and fine-tuning.

Among the 86 SNHL participants fitted with hearing aids, devices were obtained from three manufacturers. They comprised five distinct models: Phonak Audéo Infinio BTE (Phonak AG, Stäfa, Switzerland) (n = 51, 59.3%), Sonic Radiant RIC (Sonic Innovations Inc., Somerset, NJ, USA) (n = 19, 22.1%), Phonak Naída Lumity BTE (n = 6, 7.0%), Sonic Radiant BTE (n = 5, 5.8%), and Signia Silk C&G IX 7X CIC (Signia GmbH, Erlangen, Germany) (n = 5, 5.8%). By manufacturer, the distribution was Phonak 66.3% (n = 57), Sonic 27.9% (n = 24), and Signia 5.8% (n = 5). By form factor, behind-the-ear (BTE) devices accounted for 72.1% (n = 62), receiver-in-canal (RIC) for 22.1% (n = 19), and completely-in-canal (CIC) for 5.8% (n = 5). All devices were premium-level digital hearing aids with smart features, including wireless connectivity, multi-channel adaptive noise reduction, directional microphones, and smartphone application support, consistent with the inclusion criteria for the present study.

### 2.3. Assessment Instruments

Rosenberg Self-Esteem Scale (SES): The SES is a validated 10-item self-report measure of global self-worth [16]. Responses are recorded on a 4-point Likert scale (0–3), yielding total scores from 0 to 40, with higher scores indicating greater self-esteem. The instrument demonstrates excellent psychometric properties, with Cronbach’s alpha typically exceeding 0.85 [17].

Hearing Handicap Inventory for Adults (HHIA): The HHIA is a 25-item questionnaire that assesses the emotional and social/situational consequences of hearing impairment [18]. Scores range from 0 to 100, with higher scores indicating greater perceived hearing handicap. The scale classifies disability as mild (≤16), moderate (17–42), or severe (≥43) [19].

Tinnitus Handicap Inventory (THI): The THI is a 25-item instrument that quantifies the functional, emotional, and catastrophic impact of tinnitus [20]. Total scores range from 0 to 100, with severity classifications of slight (0–16), mild (18–36), moderate (38–56), severe (58–76), and catastrophic (78–100) [21].

### 2.4. Statistical Analysis

Data were analyzed using MedCalc statistical software (version 23.4.8, MedCalc Software Ltd., Ostend, Belgium), and graphs were generated in R statistical software (version 4.3.2, Vienna, Austria). Descriptive statistics included means, standard deviations, medians, and ranges. Normality of the outcome variable and of multivariate-model residuals was assessed using the Shapiro–Wilk test and visual inspection of Q–Q plots; homoscedasticity of residuals was checked using residual-vs-fitted plots; multicollinearity among the regression predictors was assessed using variance inflation factors (all VIFs < 2). Between-group comparisons were conducted using one-way analysis of variance (ANOVA) for normally distributed data and the Kruskal-Wallis H test for non-normal data. Post hoc pairwise comparisons were conducted using Tukey’s Honestly Significant Difference (HSD) test with a Bonferroni correction for multiple comparisons. Independent-samples t-tests were used to evaluate differences between specific group pairs.

Effect sizes were calculated using Cohen’s d for between-group comparisons (small: d = 0.20–0.49; medium: d = 0.50–0.79; large: d ≥ 0.80) and eta-squared (η^2^) for ANOVA (small: η^2^ = 0.01; medium: η^2^ = 0.06; large: η^2^ = 0.14) [22]. Pearson correlation coefficients were used to assess associations between continuous variables. Statistical significance was set at α = 0.05, with Bonferroni-adjusted significance levels applied for multiple comparisons (α/3 = 0.017). To further evaluate whether hearing aid use independently predicted self-esteem, a multiple linear regression was performed in the hearing-impaired cohort, stratified by hearing aid use (HA users vs. HA non-users). Self-esteem score (SES) was entered as the dependent variable, and HA use (binary), age (continuous), and sex (binary) were included as independent variables. Model fit was assessed using adjusted R^2^.

To assess potential confounding by socioeconomic factors, a sensitivity analysis was conducted among the 172 SNHL participants by sequentially adding occupational status (active vs. retired) and educational attainment (secondary vs. higher) as covariates to the primary multivariate model predicting total scores on the Rosenberg Self-Esteem Scale (RSES) from hearing-aid use, age, and sex. Among the 86 hearing-aid users, subgroup analyses compared RSES, HHIA, and THI scores by manufacturer (Phonak, Phonak AG, Stäfa, Switzerland vs. Sonic, Sonic Innovations Inc., Somerset, NJ, USA; Signia, Signia GmbH, Erlangen, Germany reported descriptively, n = 5) and by form factor (BTE vs. RIC; CIC reported descriptively, n = 5) using Welch’s *t*-tests.

## 3. Results

### 3.1. Demographic and Clinical Characteristics

The study included 245 participants divided into three groups. Demographic data and clinical scale scores are shown in Table 1. The NH control group was significantly younger than the hearing-impaired groups (mean age: 42.59 ± 13.78 years vs. 71.38 ± 12.22 and 70.28 ± 11.70 years for the HA user and HA non-user groups, respectively; *p* < 0.001). Sex distribution was balanced across groups, with a slight female predominance in the HA user group (60.5% female).

**Figure 1:**
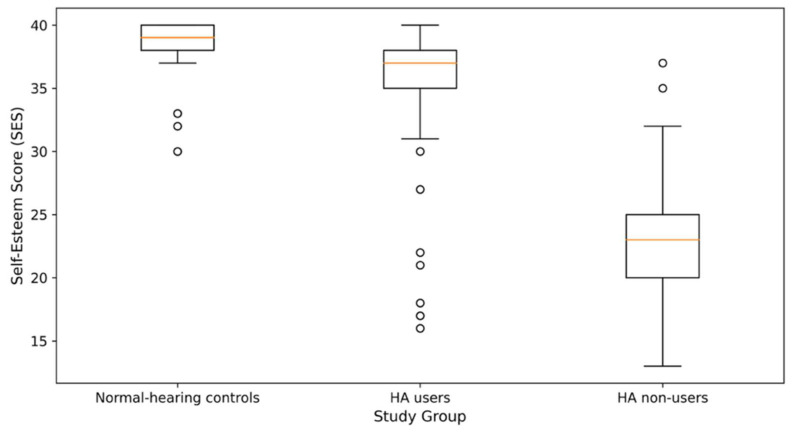
Distribution of Rosenberg Self-Esteem Scale (SES) scores across the three study groups (NH, HA users, HA non-users).

The distribution of SES scores across study groups is shown in Figure 1, which clearly separates HA users from non-users. Boxes represent the interquartile range (Q1–Q3), the horizontal line within each box is the median, and the whiskers extend to the minimum and maximum values within 1.5 × IQR. Individual participant SES scores are plotted as circles; open circles beyond the whiskers indicate outliers. HA users have substantially higher self-esteem scores than HA non-users, approaching those of NH controls.

### 3.2. Self-Esteem Outcomes

One-way ANOVA showed highly significant differences in SES scores across the three study groups (F = 299.00, *p* < 0.001, η^2^ = 0.712), indicating that group membership accounted for 71.2% of the variance in self-esteem scores (Table 2). Post hoc Tukey HSD analysis revealed that HA non-users had significantly lower self-esteem (22.99 ± 4.53) than both NH controls (38.58 ± 2.25; *p* < 0.001) and HA users (35.41 ± 5.32; *p* < 0.001). Notably, HA users’ self-esteem scores were closer to those of the NH group, although a statistically significant difference remained (*p* < 0.001).

The average difference in SES score between HA users and non-users was 12.42 points (95% CI: 10.93–13.91), indicating a large, clinically meaningful difference. An independent-samples t-test confirmed this difference (t = 16.49, *p* < 0.001) and showed a very large effect size (Cohen’s d = 2.515). These results indicate that HA use is strongly associated with higher self-esteem in adults with SNHL, although the cross-sectional design precludes causal interpretation. Detailed pairwise comparisons of SES scores, together with Cohen’s d effect sizes, are presented in Table 3.

### 3.3. Hearing Handicap and Tinnitus Outcomes

HHIA scores varied significantly across groups (F = 119.32, *p* < 0.001, η^2^ = 0.497). Normal-hearing controls showed minimal perceived handicap (6.68 ± 4.41), whereas both hearing-impaired groups reported severe disability (HA users: 49.22 ± 26.02; HA non-users: 58.42 ± 26.61). Notably, HA users reported lower HHIA scores than non-users, but this difference approached but did not reach statistical significance after Bonferroni correction (t = −2.29, *p* = 0.023; adjusted α = 0.017).

THI scores showed a similar pattern (F = 91.64, *p* < 0.001, η^2^ = 0.431), with NH controls reporting minimal tinnitus impact (6.88 ± 11.38) compared with hearing-impaired patients (HA users: 40.85 ± 21.71; HA non-users: 42.27 ± 19.43). The difference in THI scores between HA users and non-users was not statistically significant (t = −0.45, *p* = 0.652), indicating comparable tinnitus burden across groups.

### 3.4. Correlation Analysis

Pearson correlation analysis revealed significant relationships among all measured variables (Table 4). Self-esteem showed a moderate negative correlation with perceived hearing handicap (r = −0.573, *p* < 0.001), indicating that greater perceived hearing difficulty was associated with lower self-esteem. Additionally, SES showed a weaker but still significant negative correlation with tinnitus severity (r = −0.443, *p* < 0.001). Notably, HHIA and THI scores were strongly positively correlated (r = 0.729, *p* < 0.001), indicating substantial overlap between perceived hearing disability and tinnitus burden.

### 3.5. Multivariate Regression Analysis

A multiple linear regression was conducted to assess whether use of smart hearing-aid technology independently predicts self-esteem, controlling for age and sex, among hearing-impaired participants (n = 172). Diagnostic checks confirmed that all regression assumptions were met. Residuals were approximately normally distributed (Shapiro–Wilk *p* > 0.05; Q–Q plots aligned with theoretical quantiles). The residual-vs-fitted plot showed no evidence of heteroscedasticity, and all variance inflation factors were below 2. Use of smart hearing-aid technology remained a strong independent predictor of self-esteem (β = 12.38, SE = 0.76, *p* < 0.001, 95% CI: 10.88–13.88). Age (β = 0.028, *p* = 0.378) and sex (β = 0.121, *p* = 0.875) were not significant predictors. The overall model explained 61.7% of the variance in self-esteem scores (adjusted R^2^ = 0.610).

In a second model, the fully adjusted regression included smart hearing aid use, age, sex, HHIA, and THI. HA use remained a strong independent predictor of self-esteem (β = 11.87, SE = 0.74, *p* < 0.001, 95% CI: 10.42–13.33). Higher HHIA scores were independently associated with lower self-esteem (β = −0.056, *p* = 0.001), whereas tinnitus severity was not a significant independent predictor (*p* = 0.623). The model explained 65.6% of the variance in self-esteem scores (adjusted R^2^ = 0.646). The regression coefficients and 95% confidence intervals for the fully adjusted model (Model 2) are summarized in Table 5.

**Figure 2:**
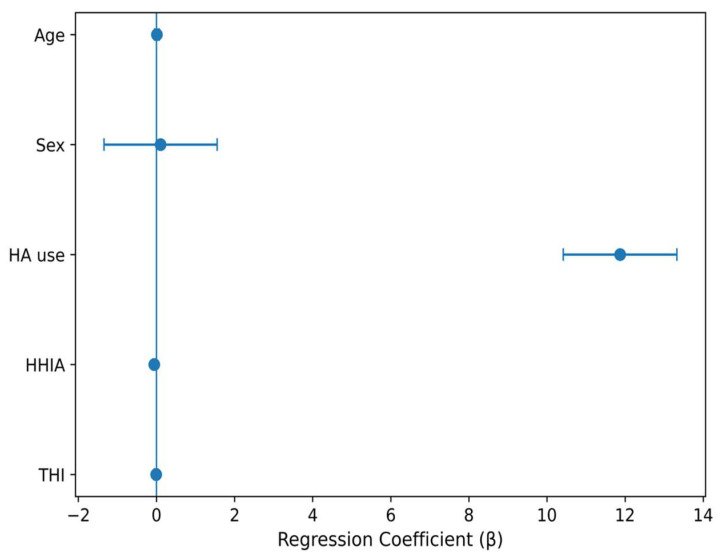
Multivariate regression coefficients (β) with 95% confidence intervals for predictors of self-esteem (SES) in the hearing-impaired cohort.

The magnitudes and directions of the regression coefficients are summarized in Figure 2. Bars to the right of zero (positive coefficients) indicate predictors associated with higher SES; bars to the left (negative coefficients) indicate predictors associated with lower SES. HA use is coded 1 = user, 0 = non-user; sex is coded 1 = female, 0 = male, so the corresponding coefficients represent the adjusted difference in SES between the two categories. Age, HHIA, and THI are continuous predictors; their coefficients represent the change in SES per one-unit increase. HA use shows a large positive coefficient (β ≈ +11.9; *p* < 0.001), whereas perceived hearing handicap (HHIA) shows a small but significant negative coefficient (β = −0.056; *p* = 0.001). Age, sex, and THI were not significant independent predictors.

### 3.6. Sensitivity Analysis

The protective association between hearing-aid use and self-esteem in the SNHL cohort (n = 172) remained essentially unchanged across all sequentially adjusted multivariate models. In the unadjusted model, hearing-aid use was associated with a 12.42-point higher RSES score (β = 12.42, SE = 0.75, t = 16.49, *p* < 0.001; R^2^ = 0.615). After adjusting for age and sex, the coefficient was 12.38 (*p* < 0.001). Sequential addition of occupational status (β_HA = 12.35, *p* < 0.001; R^2^ = 0.634) and educational attainment (β_HA = 12.45, *p* < 0.001) did not meaningfully attenuate the hearing-aid effect, indicating that the observed association is not confounded by the available socioeconomic variables. Notably, occupational status independently predicted self-esteem within the SNHL cohort: active workers reported lower RSES scores than retired participants, on average by 3.96 points (β = −3.96, SE = 1.45, *p* = 0.007), an effect that may reflect heightened workplace stigma or communication-related stress among working-age individuals with hearing impairment. Educational attainment did not independently predict RSES (*p* = 0.615), likely reflecting the low variability of this variable in the present rural cohort.

### 3.7. Hearing-Aid Subgroup Analyses

Among the 86 hearing-aid users, RSES scores did not differ significantly between Phonak (n = 57; 34.86 ± 6.10) and Sonic (n = 24; 36.08 ± 3.22) devices (t = −1.17, df = 74.9, *p* = 0.244, Cohen’s d = −0.23). Signia devices (n = 5; 38.40 ± 1.14) were reported descriptively only because of the small subgroup size. By form factor, a trend toward higher self-esteem was observed with receiver-in-canal (RIC, n = 19; 36.63 ± 2.54) compared with behind-the-ear (BTE, n = 62; 34.79 ± 5.98) devices, but the difference did not reach statistical significance (t = −1.92, df = 70.8, *p* = 0.059, d = −0.34). Hearing-handicap (HHIA) and tinnitus-handicap (THI) scores did not differ significantly across manufacturers (all *p* > 0.4). These findings suggest that the self-esteem benefit of smart-hearing-aid use is broadly consistent across manufacturers and form factors represented in this cohort, although modest subgroup sizes, particularly for Signia and CIC devices, preclude definitive device-specific conclusions.

## 4. Discussion

This study demonstrates a strong, independent statistical association between the use of smart hearing-aid technology and higher self-esteem among adults with SNHL. The between-group contrast (Cohen’s d = 2.515) exceeds conventional thresholds for a large effect and remains robust across sensitivity analyses controlling for age, HHIA, and THI. Because the design is cross-sectional, we interpret this finding as an association rather than a direct therapeutic effect. The present findings are consistent with and extend earlier work on the psychosocial benefits of hearing amplification [23,24], as well as the non-auditory gains reported more recently by the ACHIEVE randomized trial [8] and the 2024 Lancet Commission review [9]. In contrast to most prior reports, which have focused on depression, loneliness, or generic health-related quality of life, the present work specifically isolates global self-esteem—a relatively stable, trait-like construct—thereby complementing rather than duplicating the existing literature.

The markedly lower self-esteem observed in untreated hearing-impaired patients (SES mean: 22.99) compared with normal-hearing controls (SES mean: 38.58) supports theoretical models suggesting that communication difficulties and social isolation diminish self-perception [25]. Importantly, hearing aid users achieved self-esteem scores (35.41) comparable to those of individuals with normal hearing, suggesting that modern hearing technologies may help reduce the psychological effects of long-term hearing loss. This finding supports the view of hearing aids as therapeutic tools that address not only sensory deficits but also broader psychosocial issues [26].

The moderate negative correlation between self-esteem and perceived hearing handicap (r = −0.573) highlights the psychological processes underlying these findings. Patients who perceive themselves as more hearing impaired tend to have lower self-esteem, likely due to reduced social involvement, communication frustration, and negative self-assessments [27]. The finding that hearing-aid users reported lower HHIA scores than non-users, although not statistically significant after correction, suggests that assistive technology may reduce feelings of disability and, indirectly, help maintain self-esteem [28].

Contemporary hearing aids have evolved into sophisticated, smart wearables with multiple technological features aligned with principles of chronic disease management [29]. Digital signal processing algorithms enhance speech clarity across diverse listening environments, while wireless connectivity enables seamless integration with smartphones, televisions, and public assistive listening systems [30]. Advanced models include artificial intelligence-driven environmental classification, real-time translation, and biometric monitoring, placing hearing aids within the broader ecosystem of health-focused smart technologies [31]. These features collectively improve the user experience and may contribute to the psychological benefits observed in the current study.

The strong positive correlation between HHIA and THI scores (r = 0.729) indicates substantial comorbidity between perceived hearing disability and tinnitus burden, consistent with the high prevalence of tinnitus among individuals with hearing impairment [32]. However, hearing aid use did not significantly reduce THI scores compared with non-users, suggesting that although acoustic amplification may provide some tinnitus-masking benefits, additional targeted interventions may be necessary for comprehensive tinnitus management [33]. Future research should examine whether specialized hearing aid programs that incorporate sound therapy or tinnitus-specific features yield better outcomes for this patient group.

These findings have important implications for clinical practice and healthcare policy in the management of chronic diseases. The clear psychological benefits of hearing aid use support early intervention and wider access to audiological rehabilitation services [34]. Healthcare providers should routinely evaluate self-esteem and psychosocial health in patients with hearing loss, understanding that untreated hearing issues can lead to broader mental health problems [35]. Additionally, incorporating hearing aids into chronic disease management plans is justified, as they can improve both sensory function and psychological well-being.

An unanticipated finding from the sensitivity analysis was that, within the SNHL cohort, occupationally active participants reported lower self-esteem than retired participants after adjusting for age, sex, and hearing-aid use (β = −3.96, *p* = 0.007). Although the present study cannot establish causality, this observation aligns with prior reports linking working-age hearing impairment to workplace communication difficulties, perceived stigma, and reduced occupational self-efficacy [36]. It also suggests that screening and early intervention for self-esteem and psychosocial well-being may be particularly important for working-age SNHL patients, a subgroup whose needs may differ from those of older, retired patients, for whom hearing-aid use has been studied more often.

Several limitations temper the interpretation of our findings. First, the cross-sectional design precludes causal inference, and leaves open the possibility of reverse causation, that is, individuals with higher baseline self-esteem may be more likely to adopt and adhere to hearing-aid use. Second, although HA users and non-users were closely age-matched within the hearing-impaired cohort, normal-hearing controls were younger; sensitivity analyses confirmed that this imbalance did not drive the principal finding, but residual age-related effects cannot be fully excluded. Third, although occupational status and educational attainment could be retrieved retrospectively from the clinical records and incorporated into the multivariate model, several other socio-economic and psychosocial variables, including household income, health literacy, perceived stigma of hearing loss, and mental-health comorbidities, were not available; residual confounding from these factors and selection bias affecting the HA non-user group cannot be excluded. Generalizability of the present results is therefore limited to demographically comparable populations of rural, publicly insured (CAS-funded) patients accessing SNHL rehabilitation through the Romanian national insurance system; replication in urban and privately insured cohorts is warranted. Fourth, the RSES measures global self-worth and is not tailored to deaf identity or communication-specific self-perception; future studies could complement it with deaf-culture-informed instruments that assess communication quality, self-perception as a deaf or hard-of-hearing person, and community belonging. Fifth, the study was conducted at a single tertiary center; multicenter replication is required to establish generalizability. Several socio-economic variables in this cohort exhibited limited variability, which constrained the depth of confounder adjustment. All hearing-aid recipients were CAS-funded and resided in rural areas of western Romania, and educational attainment was predominantly secondary (94.7% high school), preventing meaningful analysis of income-related, urban–rural, and education-related effects on self-esteem outcomes. While occupational status could be incorporated into the multivariate model and did not attenuate the hearing-aid effect, residual confounding by unmeasured socio-economic determinants (e.g., household income, social network density, religiosity) cannot be excluded. Generalizability of these findings is therefore limited to demographically comparable populations: rural, publicly insured patients accessing SNHL rehabilitation through the national insurance system. Replication in urban and privately insured cohorts is warranted. Although specific hearing-aid models were documented, the subgroup sizes for individual manufacturers (Signia, n = 5) and form factors (CIC, n = 5) precluded formal statistical comparison of device-specific outcomes. The manufacturer- and form-factor-stratified analyses presented should therefore be regarded as exploratory; adequately powered comparative-effectiveness studies of smart-hearing-aid devices remain a priority for future work.

Future research should prioritize longitudinal designs with repeated self-esteem measurements before and after hearing-aid fitting, as well as interventional trials randomizing newly diagnosed patients to early versus delayed amplification, to establish temporal precedence between device use and psychological change. Multicenter studies that incorporate socioeconomic and educational covariates and enroll comparator groups matched by age or propensity would further reduce residual confounding. Mechanistic sub-analyses of specific smart-device features, e.g., directional microphones, AI-driven environmental classification, Bluetooth connectivity with smartphone applications, and integrated tinnitus-masking programs, would help identify which technological components contribute most to the psychosocial gains observed here. Qualitative and mixed-methods studies that incorporate perspectives from the deaf and hard-of-hearing community would further contextualize psychological outcomes beyond those captured by audiological metrics alone.

## 5. Conclusions

This cross-sectional study demonstrates a strong, independent statistical association between the use of smart hearing-aid technology and higher self-esteem in adults with sensorineural hearing loss. The magnitude of this association, which persisted after adjustment for age, sex, perceived hearing handicap, and tinnitus severity, and across age, sex, and socioeconomic-adjusted sensitivity analyses, supports the psychosocial relevance of audiological rehabilitation but does not establish hearing-aid use as a stand-alone determinant of self-esteem. Self-esteem is multifactorial and shaped by communication quality, social support, identity, employment, and mental health comorbidities not directly assessed here. Perceived hearing handicap showed a moderate negative correlation with self-esteem, highlighting the importance of addressing perceptions of subjective disability in clinical care. These findings support early access to audiological services, routine psychosocial assessment of adults with SNHL, and the inclusion of smart hearing aids among evidence-based components of long-term condition management. Longitudinal and interventional studies are needed to establish causal relationships and identify patient subgroups most likely to benefit from smart assistive technology.

## Figures and Tables

**Table 1:** Demographic characteristics and clinical scale scores.

Variable	NH Controls (n = 73)	SNHL Without HA (n = 86)	SNHL with HA (n = 86)	*p*-Value
Age, years (mean ± SD)	42.59 ± 13.78	70.28 ± 11.70	71.38 ± 12.22	<0.001
Age, years (median, IQR)	41 (34–52)	71.5 (63–80)	73 (66–79)	
Sex, n (%)				0.402
Female	37 (50.7)	45 (52.3)	52 (60.5)	
Male	36 (49.3)	41 (47.7)	34 (39.5)	
Education, n (%)				0.024
Secondary (high school)	67 (91.8)	86 (100.0)	79 (91.9)	
Higher/professional	6 (8.2)	0 (0.0)	7 (8.1)	
Occupational status, n (%)				<0.001
Active	69 (94.5)	16 (18.6)	13 (15.1)	
Retired	4 (5.5)	70 (81.4)	73 (84.9)	
Payment, n (%)				—
CAS (national insurance)	73 (100.0)	86 (100.0)	86 (100.0)	
Environment, n (%)				—
Rural	73 (100.0)	86 (100.0)	86 (100.0)	
HHIA total (mean ± SD)	6.68 ± 4.41	58.42 ± 26.61	49.22 ± 26.02	<0.001
THI total (mean ± SD)	6.88 ± 11.38	42.27 ± 19.43	40.85 ± 21.71	<0.001
RSES (self-esteem) (mean ± SD)	38.58 ± 2.25	22.99 ± 4.53	35.41 ± 5.32	<0.001

Note: *p*-values: ANOVA for continuous variables; chi-square for categorical variables. ‘—’ indicates a constant variable (zero variance) that cannot be tested. The two SNHL groups are well age-matched (*p* = 0.546 for HA vs. no-HA pairwise comparison). Abbreviations: NH, Normal Hearing; HA, Hearing Aid; SES, Self-Esteem Scale; HHIA, Hearing Handicap Inventory for Adults; THI, Tinnitus Handicap Inventory; SD, Standard Deviation.

**Table 2:** One-way ANOVA and effect size statistics.

Variable	F-Statistic	*p*-Value	η^2^ (Eta-Squared)
SES	299.00	<0.001	0.712 (Large)
HHIA	119.32	<0.001	0.497 (Large)
THI	91.64	<0.001	0.431 (Large)

Abbreviations: SES, Self-Esteem Scale; HHIA, Hearing Handicap Inventory for Adults; THI, Tinnitus Handicap Inventory.

**Table 3:** Pairwise comparisons with Cohen’s d effect sizes for SES.

Comparison	t-Statistic	*p*-Value	Cohen’s d (Interpretation)
NH vs. HA users	4.74	<0.001	0.755 (Medium)
NH vs. HA non-users	26.75	<0.001	4.257 (Very Large)
HA users vs. HA non-users	16.49	<0.001	2.515 (Very Large)

Abbreviations: NH, Normal Hearing; HA, Hearing Aid.

**Table 4:** Pearson correlation matrix.

Variable	SES	HHIA	THI
SES	1.000	−0.573 ***	−0.443 ***
HHIA	−0.573 ***	1.000	0.729 ***
THI	−0.443 ***	0.729 ***	1.000

*** *p* < 0.001.

**Table 5:** Multivariate linear regression analysis predicting self-esteem.

Predictor	Beta (β)	SE	*p*-Value	95% CI Lower	95% CI Upper
Age	0.012	0.031	0.705	−0.049	0.072
Sex	0.106	0.734	0.886	−1.343	1.554
HA use	11.872	0.736	<0.001	10.418	13.326
HHIA	−0.056	0.016	0.001	−0.087	−0.024
THI	−0.01	0.021	0.623	−0.051	0.031

Abbreviations: HA, Hearing Aid; HHIA, Hearing Handicap Inventory for Adults; THI, Tinnitus Handicap Inventory.

## Data Availability

The data presented in this study are available on request from the corresponding author. Access is restricted because the dataset contains sensitive patient health information and is subject to the ethical approval conditions granted by the Institutional Ethics Committee, which limits open dissemination to protect participant confidentiality. Requests for data access may be directed to the corresponding authors.
